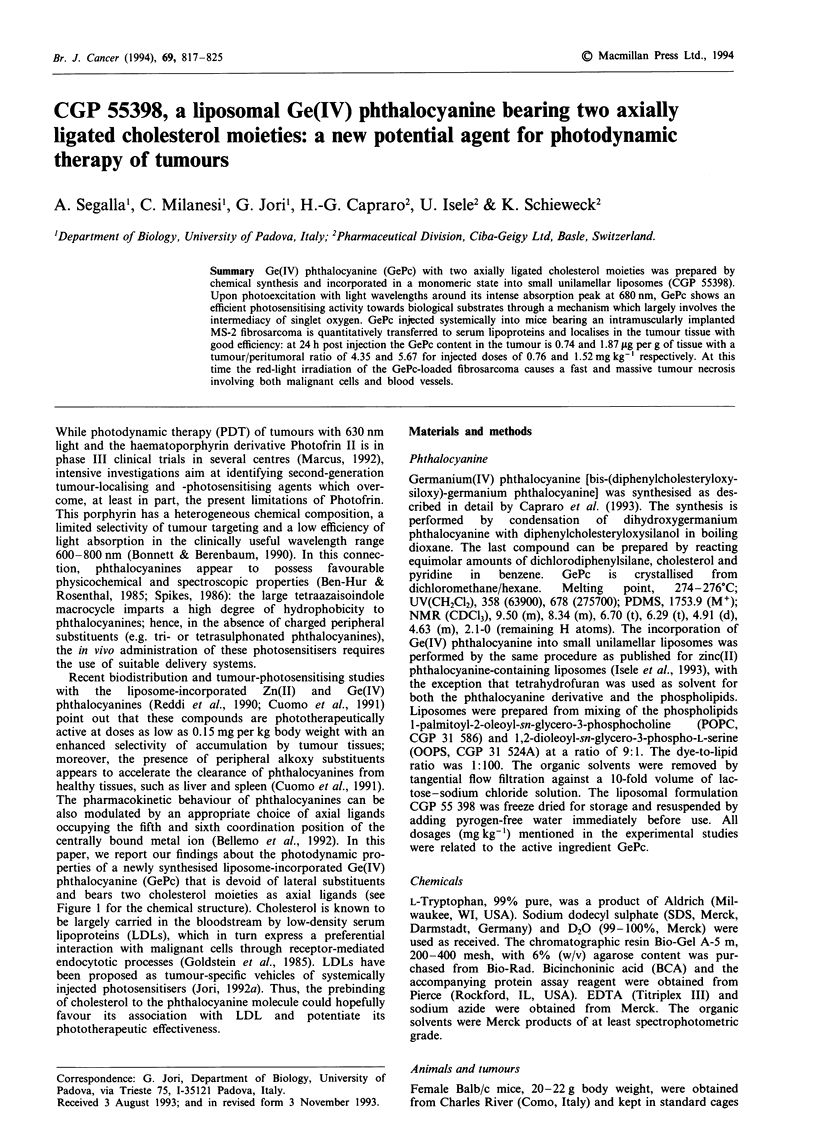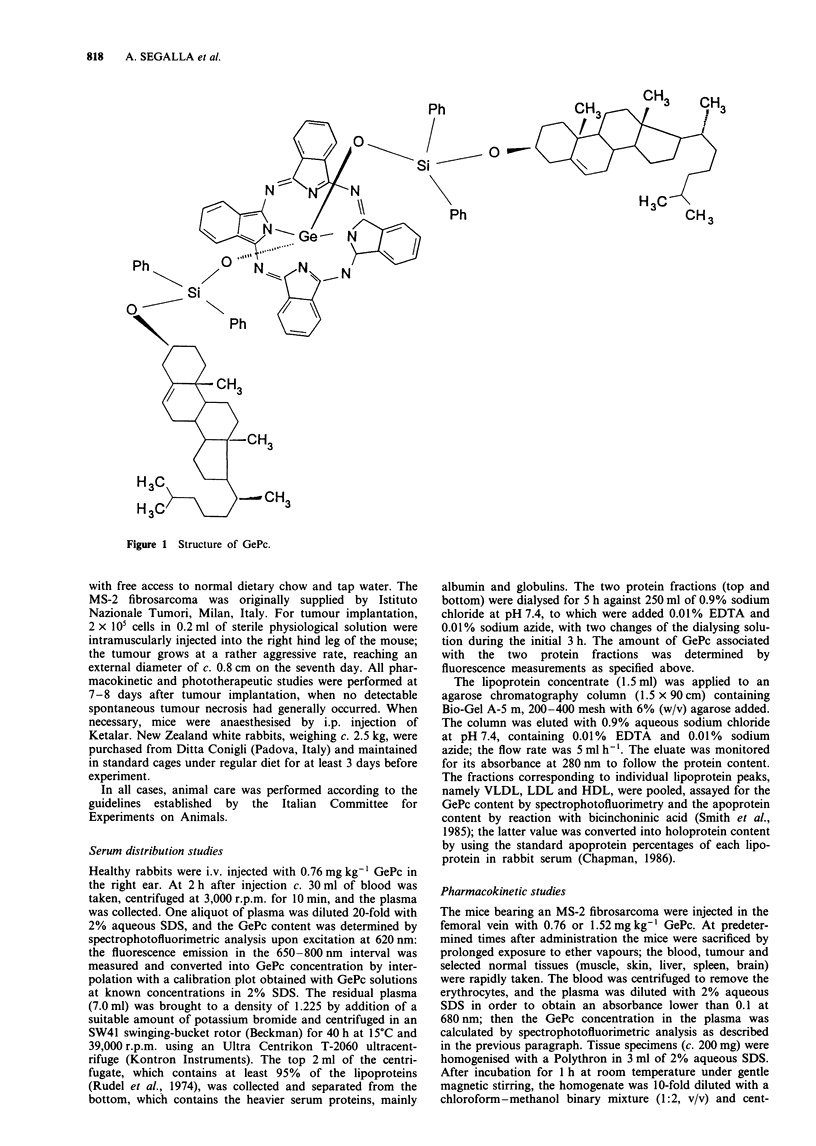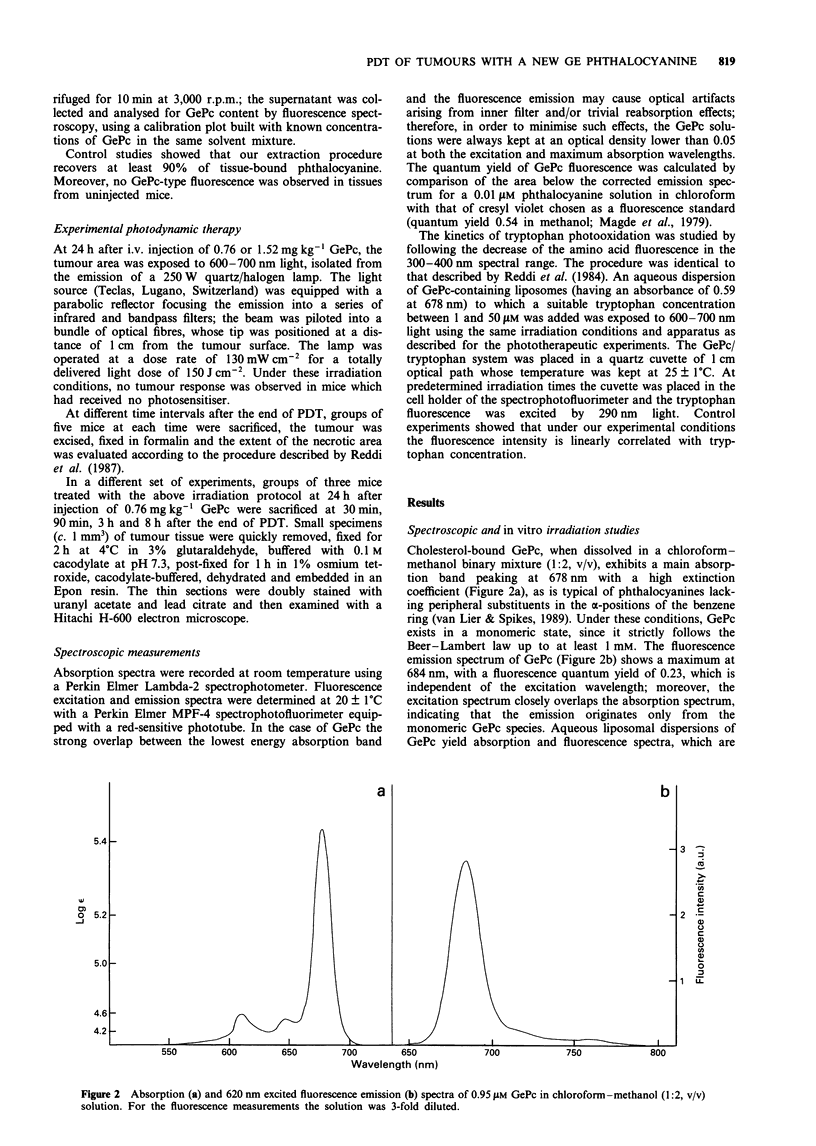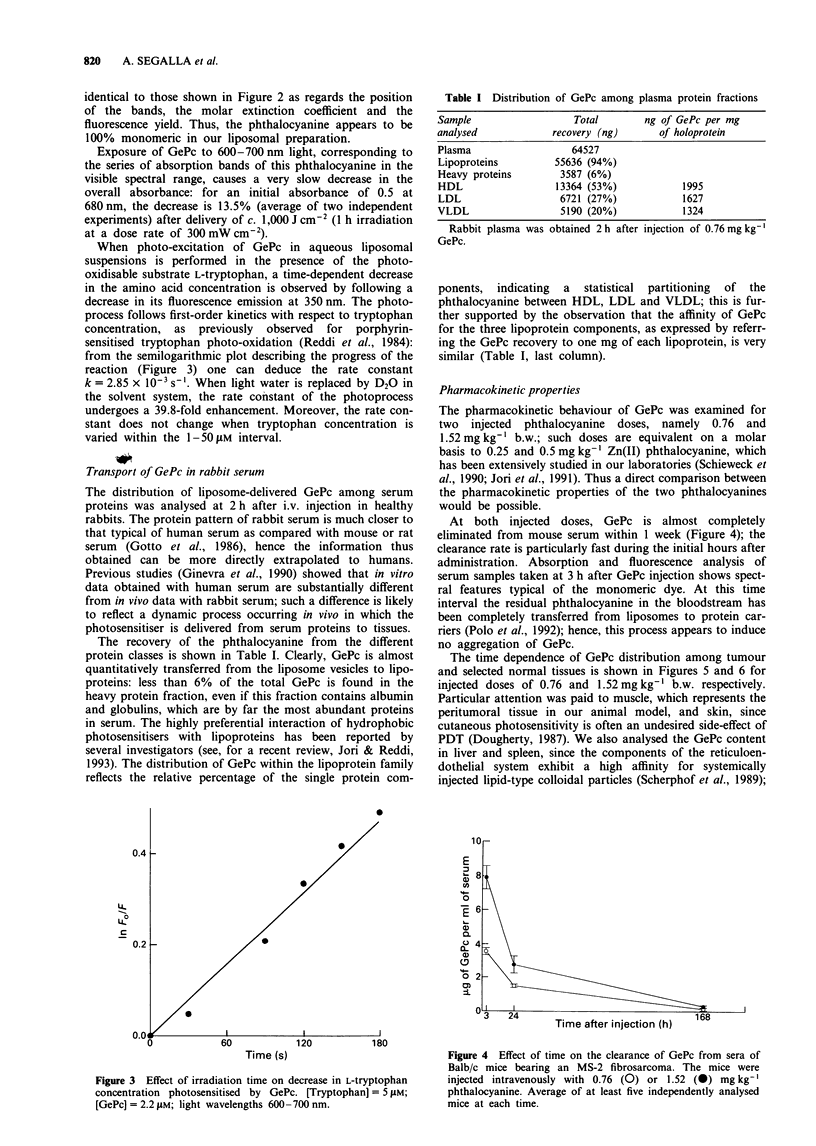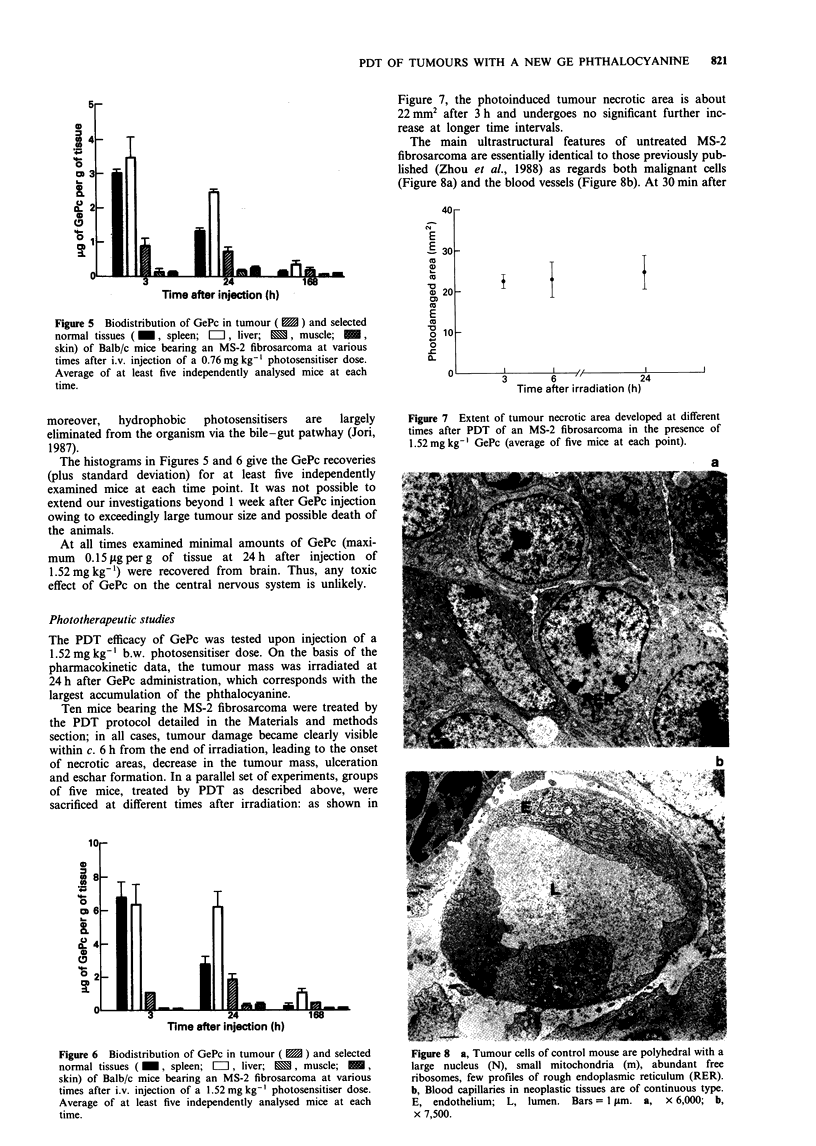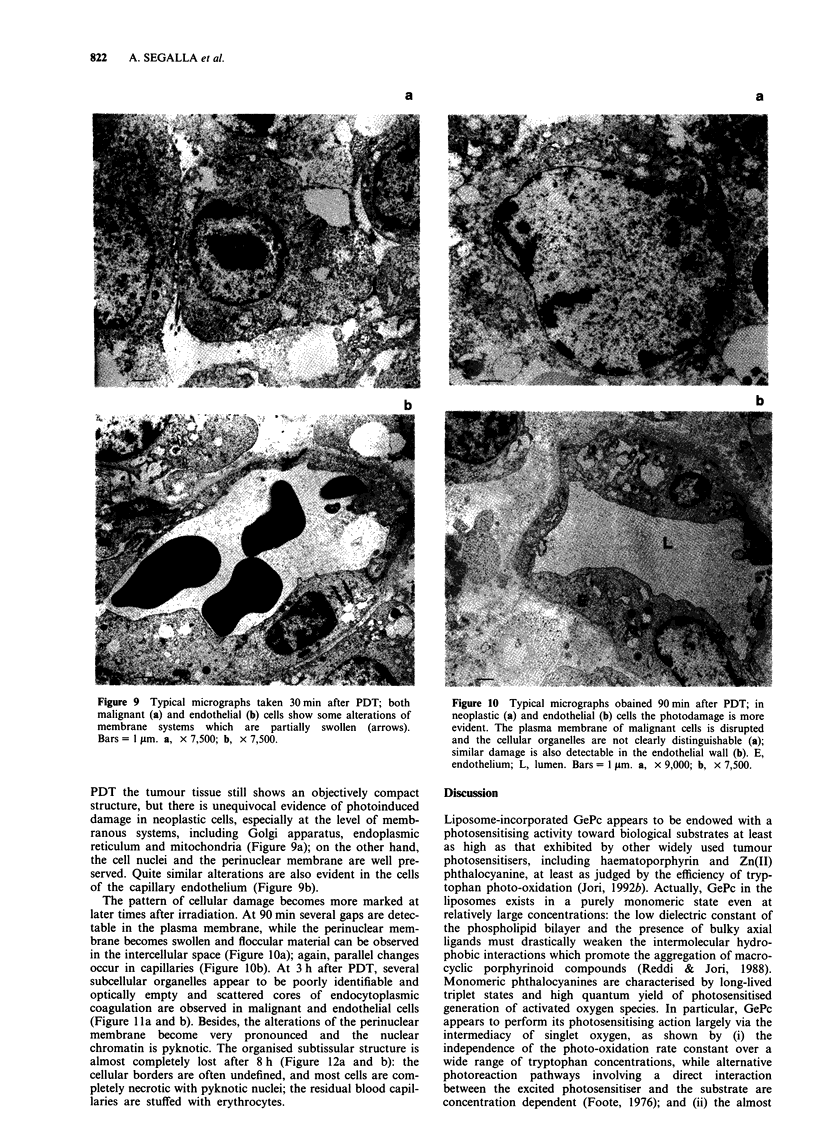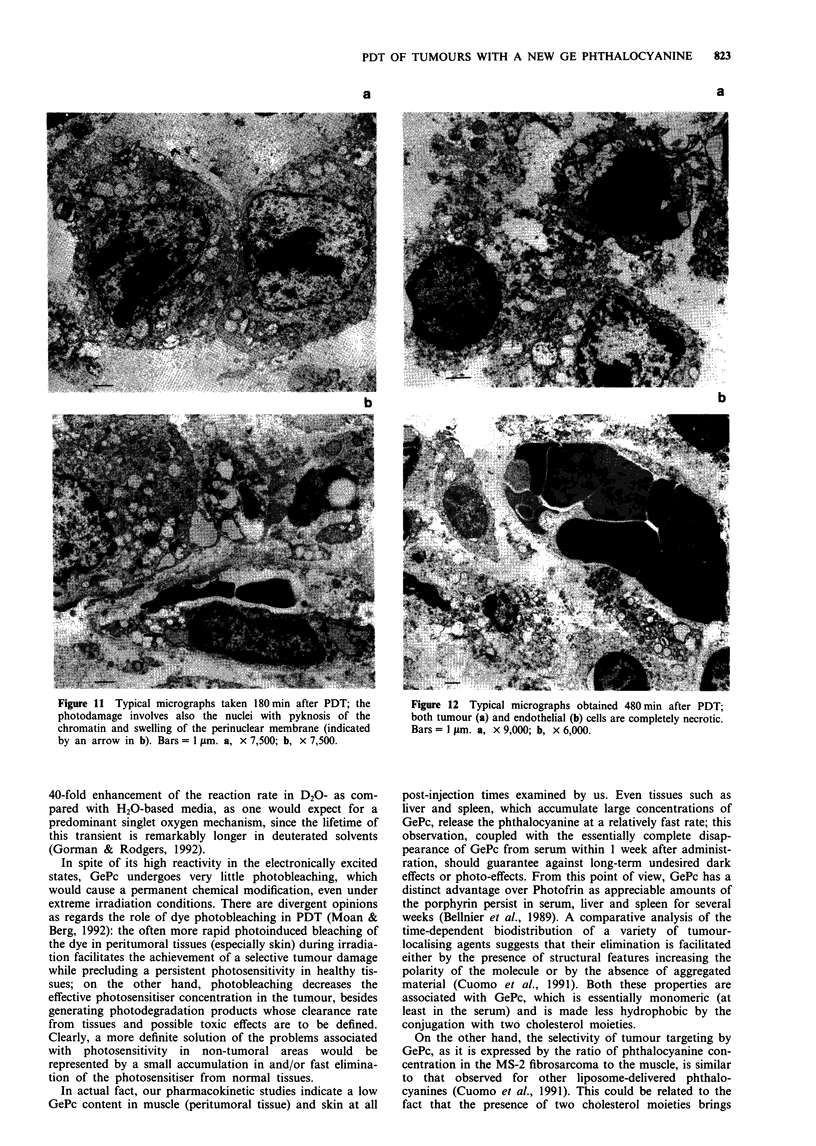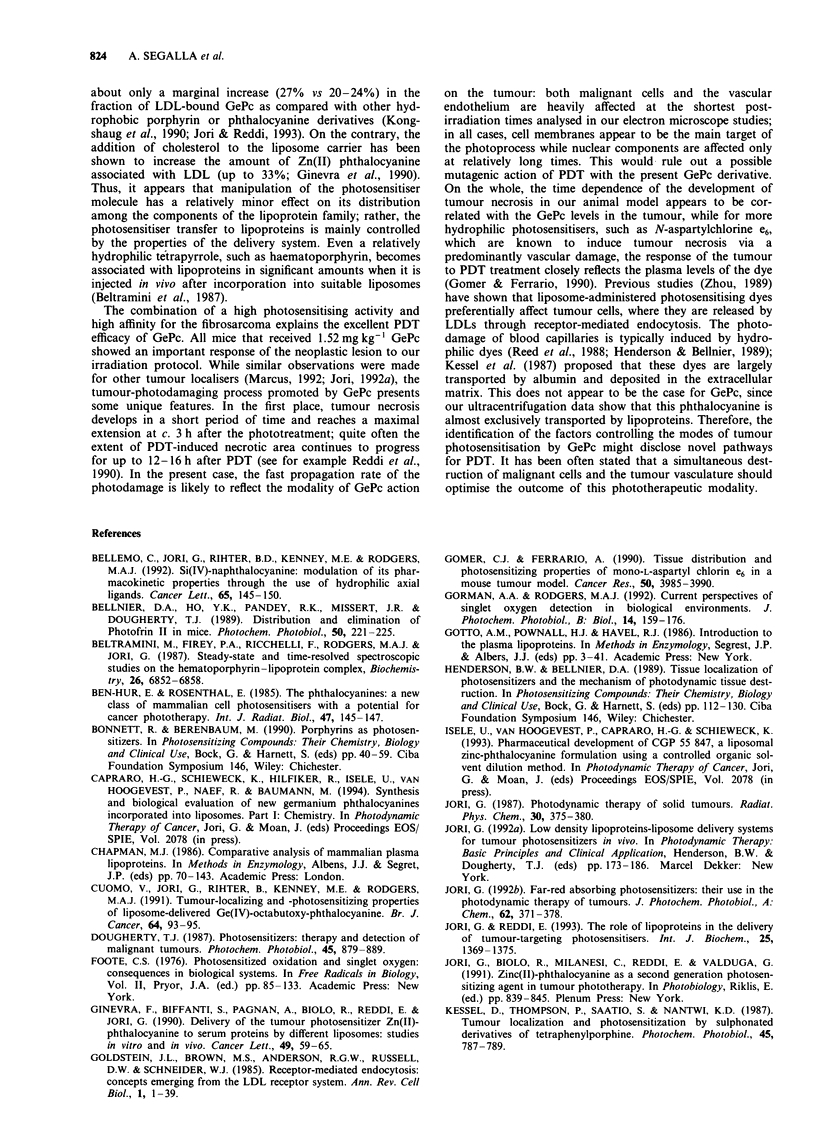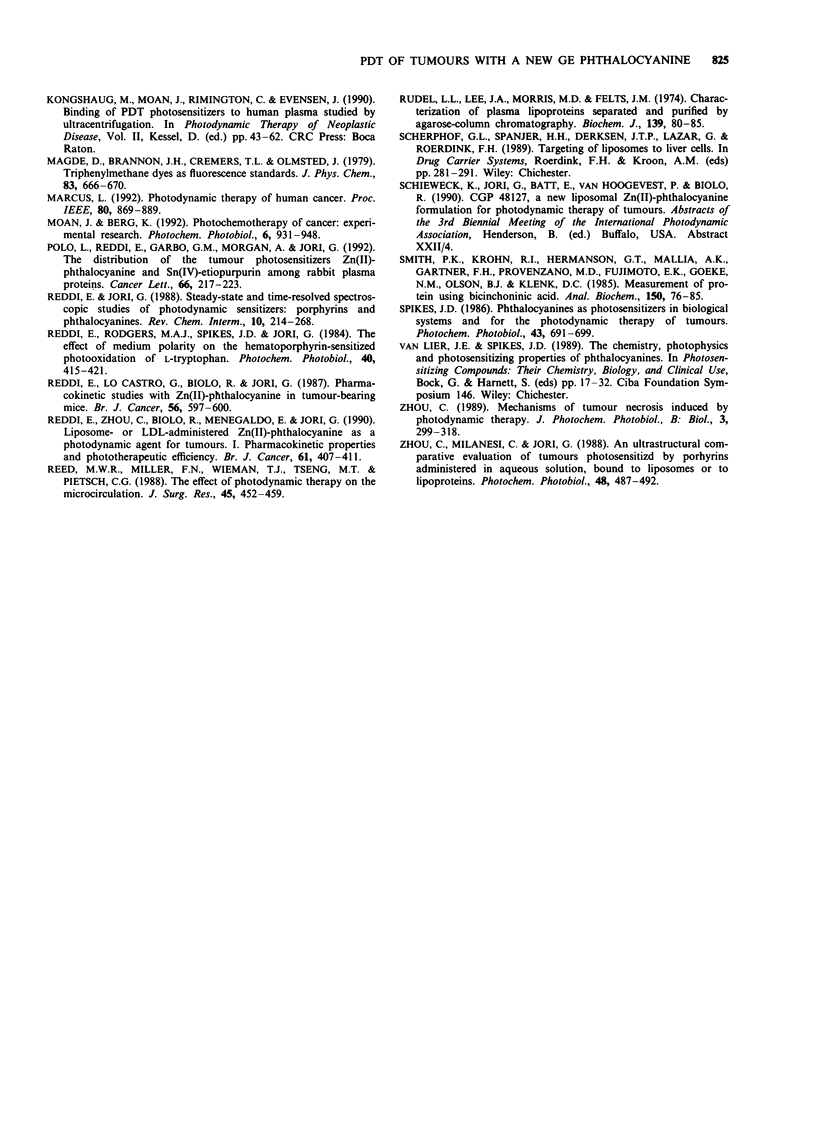# CGP 55398, a liposomal Ge(IV) phthalocyanine bearing two axially ligated cholesterol moieties: a new potential agent for photodynamic therapy of tumours.

**DOI:** 10.1038/bjc.1994.160

**Published:** 1994-05

**Authors:** A. Segalla, C. Milanesi, G. Jori, H. G. Capraro, U. Isele, K. Schieweck

**Affiliations:** Department of Biology, University of Padova, Italy.

## Abstract

**Images:**


Br. J. Cancer (1994), 69, 817–825 © Macmillan Press Ltd., 1994

### CGP 55398, a liposomal Ge(IV) phthalocyanine bearing two axially ligated cholesterol moieties: a new potential agent for photodynamic therapy of tumours

A. Segalla[1], C. Milanesi[1], G. Jori[1], H.-G. Capraro[2], U. Isele[2] & K. Schieweck[2]

[1]*Department of Biology, University of Padova, Italy;* [2]*Pharmaceutical Division, Ciba-Geigy Ltd, Basle, Switzerland.*

**Summary** Ge(IV) phthalocyanine (GePc) with two axially ligated cholesterol moieties was prepared by chemical synthesis and incorporated in a monomeric state into small unilamellar liposomes (CGP 55398). Upon photoexcitation with light wavelengths around its intense absorption peak at 680 nm, GePc shows an efficient photosensitising activity towards biological substrates through a mechanism which largely involves the intermediacy of singlet oxygen. GePc injected systemically into mice bearing an intramuscularly implanted MS-2 fibrosarcoma is quantitatively transferred to serum lipoproteins and localises in the tumour tissue with good efficiency: at 24 h post injection the GePc content in the tumour is 0.74 and 1.87 μg per g of tissue with a tumour/peritumoral ratio of 4.35 and 5.67 for injected doses of 0.76 and 1.52 mg $kg^{-1}$ respectively. At this time the red-light irradiation of the GePc-loaded fibrosarcoma causes a fast and massive tumour necrosis involving both malignant cells and blood vessels.

While photodynamic therapy (PDT) of tumours with 630 nm light and the haematoporphyrin derivative Photofrin II is in phase III clinical trials in several centres (Marcus, 1992), intensive investigations aim at identifying second-generation tumour-localising and -photosensitising agents which overcome, at least in part, the present limitations of Photofrin. This porphyrin has a heterogeneous chemical composition, a limited selectivity of tumour targeting and a low efficiency of light absorption in the clinically useful wavelength range 600–800 nm (Bonnett & Berenbaum, 1990). In this connection, phthalocyanines appear to possess favourable physicochemical and spectroscopic properties (Ben-Hur & Rosenthal, 1985; Spikes, 1986): the large tetraazaisoindole macrocycle imparts a high degree of hydrophobicity to phthalocyanines; hence, in the absence of charged peripheral substituents (e.g. tri- or tetrasulphonated phthalocyanines), the *in vivo* administration of these photosensitisers requires the use of suitable delivery systems.

Recent biodistribution and tumour-photosensitising studies with the liposome-incorporated Zn(II) and Ge(IV) phthalocyanines (Reddi *et al.*, 1990; Cuomo *et al.*, 1991) point out that these compounds are phototherapeutically active at doses as low as 0.15 mg per kg body weight with an enhanced selectivity of accumulation by tumour tissues; moreover, the presence of peripheral alkoxy substituents appears to accelerate the clearance of phthalocyanines from healthy tissues, such as liver and spleen (Cuomo *et al.*, 1991). The pharmacokinetic behaviour of phthalocyanines can be also modulated by an appropriate choice of axial ligands occupying the fifth and sixth coordination position of the centrally bound metal ion (Bellemo *et al.*, 1992). In this paper, we report our findings about the photodynamic properties of a newly synthesised liposome-incorporated Ge(IV) phthalocyanine (GePc) that is devoid of lateral substituents and bears two cholesterol moieties as axial ligands (see Figure 1 for the chemical structure). Cholesterol is known to be largely carried in the bloodstream by low-density serum lipoproteins (LDLs), which in turn express a preferential interaction with malignant cells through receptor-mediated endocytotic processes (Goldstein *et al.*, 1985). LDLs have been proposed as tumour-specific vehicles of systemically injected photosensitisers (Jori, 1992*a*). Thus, the prebinding of cholesterol to the phthalocyanine molecule could hopefully favour its association with LDL and potentiate its phototherapeutic effectiveness.

Correspondence: G. Jori, Department of Biology, University of Padova, via Trieste 75, I-35121 Padova, Italy.
Received 3 August 1993; and in revised form 3 November 1993.

### Materials and methods

### *Phthalocyanine*

Germanium(IV) phthalocyanine [bis-(diphenylcholesteryloxy-siloxy)-germanium phthalocyanine] was synthesised as described in detail by Capraro *et al.* (1993). The synthesis is performed by condensation of dihydroxygermanium phthalocyanine with diphenylcholesteryloxysilanol in boiling dioxane. The last compound can be prepared by reacting equimolar amounts of dichlorodiphenylsilane, cholesterol and pyridine in benzene. GePc is crystallised from dichloromethane/hexane. Melting point, 274–276°C; UV($CH_2Cl_2$), 358 (63900), 678 (275700); PDMS, 1753.9 ($M^+$); NMR ($CDCl_3$), 9.50 (m), 8.34 (m), 6.70 (t), 6.29 (t), 4.91 (d), 4.63 (m), 2.1-0 (remaining H atoms). The incorporation of Ge(IV) phthalocyanine into small unilamellar liposomes was performed by the same procedure as published for zinc(II) phthalocyanine-containing liposomes (Isele *et al.*, 1993), with the exception that tetrahydrofuran was used as solvent for both the phthalocyanine derivative and the phospholipids. Liposomes were prepared from mixing of the phospholipids 1-palmitoyl-2-oleoyl-*sn*-glycero-3-phosphocholine (POPC, CGP 31 586) and 1,2-dioleoyl-*sn*-glycero-3-phospho-L-serine (OOPS, CGP 31 524A) at a ratio of 9:1. The dye-to-lipid ratio was 1:100. The organic solvents were removed by tangential flow filtration against a 10-fold volume of lactose–sodium chloride solution. The liposomal formulation CGP 55 398 was freeze dried for storage and resuspended by adding pyrogen-free water immediately before use. All dosages (mg $kg^{-1}$) mentioned in the experimental studies were related to the active ingredient GePc.

### *Chemicals*

L-Tryptophan, 99% pure, was a product of Aldrich (Milwaukee, WI, USA). Sodium dodecyl sulphate (SDS, Merck, Darmstadt, Germany) and $D_2O$ (99–100%, Merck) were used as received. The chromatographic resin Bio-Gel A-5 m, 200–400 mesh, with 6% (w/v) agarose content was purchased from Bio-Rad. Bicinchoninic acid (BCA) and the accompanying protein assay reagent were obtained from Pierce (Rockford, IL, USA). EDTA (Titriplex III) and sodium azide were obtained from Merck. The organic solvents were Merck products of at least spectrophotometric grade.

### *Animals and tumours*

Female Balb/c mice, 20–22 g body weight, were obtained from Charles River (Como, Italy) and kept in standard cages

**Figure 1** Structure of GePc.

with free access to normal dietary chow and tap water. The MS-2 fibrosarcoma was originally supplied by Istituto Nazionale Tumori, Milan, Italy. For tumour implantation, $2 \times 10^5$ cells in 0.2 ml of sterile physiological solution were intramuscularly injected into the right hind leg of the mouse; the tumour grows at a rather aggressive rate, reaching an external diameter of c. 0.8 cm on the seventh day. All pharmacokinetic and phototherapeutic studies were performed at 7–8 days after tumour implantation, when no detectable spontaneous tumour necrosis had generally occurred. When necessary, mice were anaesthesised by i.p. injection of Ketalar. New Zealand white rabbits, weighing c. 2.5 kg, were purchased from Ditta Conigli (Padova, Italy) and maintained in standard cages under regular diet for at least 3 days before experiment.

In all cases, animal care was performed according to the guidelines established by the Italian Committee for Experiments on Animals.

*Serum distribution studies*

Healthy rabbits were i.v. injected with 0.76 mg $kg^{-1}$ GePc in the right ear. At 2 h after injection c. 30 ml of blood was taken, centrifuged at 3,000 r.p.m. for 10 min, and the plasma was collected. One aliquot of plasma was diluted 20-fold with 2% aqueous SDS, and the GePc content was determined by spectrophotofluorimetric analysis upon excitation at 620 nm: the fluorescence emission in the 650–800 nm interval was measured and converted into GePc concentration by interpolation with a calibration plot obtained with GePc solutions at known concentrations in 2% SDS. The residual plasma (7.0 ml) was brought to a density of 1.225 by addition of a suitable amount of potassium bromide and centrifuged in an SW41 swinging-bucket rotor (Beckman) for 40 h at 15°C and 39,000 r.p.m. using an Ultra Centrikon T-2060 ultracentrifuge (Kontron Instruments). The top 2 ml of the centrifugate, which contains at least 95% of the lipoproteins (Rudel et al., 1974), was collected and separated from the bottom, which contains the heavier serum proteins, mainly albumin and globulins. The two protein fractions (top and bottom) were dialysed for 5 h against 250 ml of 0.9% sodium chloride at pH 7.4, to which were added 0.01% EDTA and 0.01% sodium azide, with two changes of the dialysing solution during the initial 3 h. The amount of GePc associated with the two protein fractions was determined by fluorescence measurements as specified above.

The lipoprotein concentrate (1.5 ml) was applied to an agarose chromatography column (1.5 × 90 cm) containing Bio-Gel A-5 m, 200–400 mesh with 6% (w/v) agarose added. The column was eluted with 0.9% aqueous sodium chloride at pH 7.4, containing 0.01% EDTA and 0.01% sodium azide; the flow rate was 5 ml $h^{-1}$. The eluate was monitored for its absorbance at 280 nm to follow the protein content. The fractions corresponding to individual lipoprotein peaks, namely VLDL, LDL and HDL, were pooled, assayed for the GePc content by spectrophotofluorimetry and the apoprotein content by reaction with bicinchoninic acid (Smith et al., 1985); the latter value was converted into holoprotein content by using the standard apoprotein percentages of each lipoprotein in rabbit serum (Chapman, 1986).

*Pharmacokinetic studies*

The mice bearing an MS-2 fibrosarcoma were injected in the femoral vein with 0.76 or 1.52 mg $kg^{-1}$ GePc. At predetermined times after administration the mice were sacrificed by prolonged exposure to ether vapours; the blood, tumour and selected normal tissues (muscle, skin, liver, spleen, brain) were rapidly taken. The blood was centrifuged to remove the erythrocytes, and the plasma was diluted with 2% aqueous SDS in order to obtain an absorbance lower than 0.1 at 680 nm; then the GePc concentration in the plasma was calculated by spectrophotofluorimetric analysis as described in the previous paragraph. Tissue specimens (c. 200 mg) were homogenised with a Polythron in 3 ml of 2% aqueous SDS. After incubation for 1 h at room temperature under gentle magnetic stirring, the homogenate was 10-fold diluted with a chloroform–methanol binary mixture (1:2, v/v) and centrifuged for 10 min at 3,000 r.p.m.; the supernatant was collected and analysed for GePc content by fluorescence spectroscopy, using a calibration plot built with known concentrations of GePc in the same solvent mixture.

Control studies showed that our extraction procedure recovers at least 90% of tissue-bound phthalocyanine. Moreover, no GePc-type fluorescence was observed in tissues from uninjected mice.

### *Experimental photodynamic therapy*

At 24 h after i.v. injection of 0.76 or 1.52 mg $kg^{-1}$ GePc, the tumour area was exposed to 600–700 nm light, isolated from the emission of a 250 W quartz/halogen lamp. The light source (Teclas, Lugano, Switzerland) was equipped with a parabolic reflector focusing the emission into a series of infrared and bandpass filters; the beam was piloted into a bundle of optical fibres, whose tip was positioned at a distance of 1 cm from the tumour surface. The lamp was operated at a dose rate of 130 mW $cm^{-2}$ for a totally delivered light dose of 150 J $cm^{-2}$. Under these irradiation conditions, no tumour response was observed in mice which had received no photosensitiser.

At different time intervals after the end of PDT, groups of five mice at each time were sacrificed, the tumour was excised, fixed in formalin and the extent of the necrotic area was evaluated according to the procedure described by Reddi *et al.* (1987).

In a different set of experiments, groups of three mice treated with the above irradiation protocol at 24 h after injection of 0.76 mg $kg^{-1}$ GePc were sacrificed at 30 min, 90 min, 3 h and 8 h after the end of PDT. Small specimens (*c.* 1 $mm^3$) of tumour tissue were quickly removed, fixed for 2 h at 4°C in 3% glutaraldehyde, buffered with 0.1 M cacodylate at pH 7.3, post-fixed for 1 h in 1% osmium tetroxide, cacodylate-buffered, dehydrated and embedded in an Epon resin. The thin sections were doubly stained with uranyl acetate and lead citrate and then examined with a Hitachi H-600 electron microscope.

### *Spectroscopic measurements*

Absorption spectra were recorded at room temperature using a Perkin Elmer Lambda-2 spectrophotometer. Fluorescence excitation and emission spectra were determined at 20 ± 1°C with a Perkin Elmer MPF-4 spectrophotofluorimeter equipped with a red-sensitive phototube. In the case of GePc the strong overlap between the lowest energy absorption band and the fluorescence emission may cause optical artifacts arising from inner filter and/or trivial reabsorption effects; therefore, in order to minimise such effects, the GePc solutions were always kept at an optical density lower than 0.05 at both the excitation and maximum absorption wavelengths. The quantum yield of GePc fluorescence was calculated by comparison of the area below the corrected emission spectrum for a 0.01 μM phthalocyanine solution in chloroform with that of cresyl violet chosen as a fluorescence standard (quantum yield 0.54 in methanol; Magde *et al.*, 1979).

The kinetics of tryptophan photooxidation was studied by following the decrease of the amino acid fluorescence in the 300–400 nm spectral range. The procedure was identical to that described by Reddi *et al.* (1984). An aqueous dispersion of GePc-containing liposomes (having an absorbance of 0.59 at 678 nm) to which a suitable tryptophan concentration between 1 and 50 μM was added was exposed to 600–700 nm light using the same irradiation conditions and apparatus as described for the phototherapeutic experiments. The GePc/tryptophan system was placed in a quartz cuvette of 1 cm optical path whose temperature was kept at 25 ± 1°C. At predetermined irradiation times the cuvette was placed in the cell holder of the spectrophotofluorimeter and the tryptophan fluorescence was excited by 290 nm light. Control experiments showed that under our experimental conditions the fluorescence intensity is linearly correlated with tryptophan concentration.

### Results

### *Spectroscopic and* in vitro *irradiation studies*

Cholesterol-bound GePc, when dissolved in a chloroform–methanol binary mixture (1:2, v/v), exhibits a main absorption band peaking at 678 nm with a high extinction coefficient (Figure 2a), as is typical of phthalocyanines lacking peripheral substituents in the α-positions of the benzene ring (van Lier & Spikes, 1989). Under these conditions, GePc exists in a monomeric state, since it strictly follows the Beer–Lambert law up to at least 1 mM. The fluorescence emission spectrum of GePc (Figure 2b) shows a maximum at 684 nm, with a fluorescence quantum yield of 0.23, which is independent of the excitation wavelength; moreover, the excitation spectrum closely overlaps the absorption spectrum, indicating that the emission originates only from the monomeric GePc species. Aqueous liposomal dispersions of GePc yield absorption and fluorescence spectra, which are

**Figure 2** Absorption (**a**) and 620 nm excited fluorescence emission (**b**) spectra of 0.95 μM GePc in chloroform–methanol (1:2, v/v) solution. For the fluorescence measurements the solution was 3-fold diluted.

identical to those shown in Figure 2 as regards the position of the bands, the molar extinction coefficient and the fluorescence yield. Thus, the phthalocyanine appears to be 100% monomeric in our liposomal preparation.

Exposure of GePc to 600–700 nm light, corresponding to the series of absorption bands of this phthalocyanine in the visible spectral range, causes a very slow decrease in the overall absorbance: for an initial absorbance of 0.5 at 680 nm, the decrease is 13.5% (average of two independent experiments) after delivery of *c.* 1,000 J $cm^{-2}$ (1 h irradiation at a dose rate of 300 mW $cm^{-2}$).

When photo-excitation of GePc in aqueous liposomal suspensions is performed in the presence of the photo-oxidisable substrate L-tryptophan, a time-dependent decrease in the amino acid concentration is observed by following a decrease in its fluorescence emission at 350 nm. The photo-process follows first-order kinetics with respect to tryptophan concentration, as previously observed for porphyrin-sensitised tryptophan photo-oxidation (Reddi *et al.*, 1984): from the semilogarithmic plot describing the progress of the reaction (Figure 3) one can deduce the rate constant $k = 2.85 \times 10^{-3}\ s^{-1}$. When light water is replaced by $D_2O$ in the solvent system, the rate constant of the photoprocess undergoes a 39.8-fold enhancement. Moreover, the rate constant does not change when tryptophan concentration is varied within the 1–50 µM interval.

*Transport of GePc in rabbit serum*

The distribution of liposome-delivered GePc among serum proteins was analysed at 2 h after i.v. injection in healthy rabbits. The protein pattern of rabbit serum is much closer to that typical of human serum as compared with mouse or rat serum (Gotto *et al.*, 1986), hence the information thus obtained can be more directly extrapolated to humans. Previous studies (Ginevra *et al.*, 1990) showed that *in vitro* data obtained with human serum are substantially different from *in vivo* data with rabbit serum; such a difference is likely to reflect a dynamic process occurring *in vivo* in which the photosensitiser is delivered from serum proteins to tissues.

The recovery of the phthalocyanine from the different protein classes is shown in Table I. Clearly, GePc is almost quantitatively transferred from the liposome vesicles to lipoproteins: less than 6% of the total GePc is found in the heavy protein fraction, even if this fraction contains albumin and globulins, which are by far the most abundant proteins in serum. The highly preferential interaction of hydrophobic photosensitisers with lipoproteins has been reported by several investigators (see, for a recent review, Jori & Reddi, 1993). The distribution of GePc within the lipoprotein family reflects the relative percentage of the single protein components, indicating a statistical partitioning of the phthalocyanine between HDL, LDL and VLDL; this is further supported by the observation that the affinity of GePc for the three lipoprotein components, as expressed by referring the GePc recovery to one mg of each lipoprotein, is very similar (Table I, last column).

**Table I** Distribution of GePc among plasma protein fractions

| *Sample analysed* | *Total recovery (ng)* | *ng of GePc per mg of holoprotein* |
|---|---|---|
| Plasma | 64527 | |
| Lipoproteins | 55636 (94%) | |
| Heavy proteins | 3587 (6%) | |
| HDL | 13364 (53%) | 1995 |
| LDL | 6721 (27%) | 1627 |
| VLDL | 5190 (20%) | 1324 |

Rabbit plasma was obtained 2 h after injection of 0.76 mg $kg^{-1}$ GePc.

*Pharmacokinetic properties*

The pharmacokinetic behaviour of GePc was examined for two injected phthalocyanine doses, namely 0.76 and 1.52 mg $kg^{-1}$ b.w.; such doses are equivalent on a molar basis to 0.25 and 0.5 mg $kg^{-1}$ Zn(II) phthalocyanine, which has been extensively studied in our laboratories (Schieweck *et al.*, 1990; Jori *et al.*, 1991). Thus a direct comparison between the pharmacokinetic properties of the two phthalocyanines would be possible.

At both injected doses, GePc is almost completely eliminated from mouse serum within 1 week (Figure 4); the clearance rate is particularly fast during the initial hours after administration. Absorption and fluorescence analysis of serum samples taken at 3 h after GePc injection shows spectral features typical of the monomeric dye. At this time interval the residual phthalocyanine in the bloodstream has been completely transferred from liposomes to protein carriers (Polo *et al.*, 1992); hence, this process appears to induce no aggregation of GePc.

The time dependence of GePc distribution among tumour and selected normal tissues is shown in Figures 5 and 6 for injected doses of 0.76 and 1.52 mg $kg^{-1}$ b.w. respectively. Particular attention was paid to muscle, which represents the peritumoral tissue in our animal model, and skin, since cutaneous photosensitivity is often an undesired side-effect of PDT (Dougherty, 1987). We also analysed the GePc content in liver and spleen, since the components of the reticuloendothelial system exhibit a high affinity for systemically injected lipid-type colloidal particles (Scherphof *et al.*, 1989);

**Figure 3** Effect of irradiation time on decrease in L-tryptophan concentration photosensitised by GePc. [Tryptophan] = 5 µM; [GePc] = 2.2 µM; light wavelengths 600–700 nm.

**Figure 4** Effect of time on the clearance of GePc from sera of Balb/c mice bearing an MS-2 fibrosarcoma. The mice were injected intravenously with 0.76 (○) or 1.52 (●) mg $kg^{-1}$ phthalocyanine. Average of at least five independently analysed mice at each time.

**Figure 5** Biodistribution of GePc in tumour ( ▨ ) and selected normal tissues ( ■ , spleen; □ , liver; ▧ , muscle; ▦ , skin) of Balb/c mice bearing an MS-2 fibrosarcoma at various times after i.v. injection of a 0.76 mg kg$^{-1}$ photosensitiser dose. Average of at least five independently analysed mice at each time.

moreover, hydrophobic photosensitisers are largely eliminated from the organism via the bile-gut patwhay (Jori, 1987).

The histograms in Figures 5 and 6 give the GePc recoveries (plus standard deviation) for at least five independently examined mice at each time point. It was not possible to extend our investigations beyond 1 week after GePc injection owing to exceedingly large tumour size and possible death of the animals.

At all times examined minimal amounts of GePc (maximum 0.15 µg per g of tissue at 24 h after injection of 1.52 mg kg$^{-1}$) were recovered from brain. Thus, any toxic effect of GePc on the central nervous system is unlikely.

*Phototherapeutic studies*

The PDT efficacy of GePc was tested upon injection of a 1.52 mg kg$^{-1}$ b.w. photosensitiser dose. On the basis of the pharmacokinetic data, the tumour mass was irradiated at 24 h after GePc administration, which corresponds with the largest accumulation of the phthalocyanine.

Ten mice bearing the MS-2 fibrosarcoma were treated by the PDT protocol detailed in the Materials and methods section; in all cases, tumour damage became clearly visible within *c.* 6 h from the end of irradiation, leading to the onset of necrotic areas, decrease in the tumour mass, ulceration and eschar formation. In a parallel set of experiments, groups of five mice, treated by PDT as described above, were sacrificed at different times after irradiation: as shown in Figure 7, the photoinduced tumour necrotic area is about 22 mm$^2$ after 3 h and undergoes no significant further increase at longer time intervals.

The main ultrastructural features of untreated MS-2 fibrosarcoma are essentially identical to those previously published (Zhou *et al.*, 1988) as regards both malignant cells (Figure 8a) and the blood vessels (Figure 8b). At 30 min after

**Figure 7** Extent of tumour necrotic area developed at different times after PDT of an MS-2 fibrosarcoma in the presence of 1.52 mg kg$^{-1}$ GePc (average of five mice at each point).

**Figure 8** **a**, Tumour cells of control mouse are polyhedral with a large nucleus (N), small mitochondria (m), abundant free ribosomes, few profiles of rough endoplasmic reticulum (RER). **b**, Blood capillaries in neoplastic tissues are of continuous type. E, endothelium; L, lumen. Bars = 1 µm. **a**, × 6,000; **b**, × 7,500.

**Figure 6** Biodistribution of GePc in tumour ( ▨ ) and selected normal tissues ( ■ , spleen; □ , liver; ▧ , muscle; ▦ , skin) of Balb/c mice bearing an MS-2 fibrosarcoma at various times after i.v. injection of a 1.52 mg kg$^{-1}$ photosensitiser dose. Average of at least five independently analysed mice at each time.

**Figure 9** Typical micrographs taken 30 min after PDT; both malignant (**a**) and endothelial (**b**) cells show some alterations of membrane systems which are partially swollen (arrows). Bars = 1 μm. **a**, × 7,500; **b**, × 7,500.

**Figure 10** Typical micrographs obained 90 min after PDT; in neoplastic (**a**) and endothelial (**b**) cells the photodamage is more evident. The plasma membrane of malignant cells is disrupted and the cellular organelles are not clearly distinguishable (**a**); similar damage is also detectable in the endothelial wall (**b**). E, endothelium; L, lumen. Bars = 1 μm. **a**, × 9,000; **b**, × 7,500.

PDT the tumour tissue still shows an objectively compact structure, but there is unequivocal evidence of photoinduced damage in neoplastic cells, especially at the level of membranous systems, including Golgi apparatus, endoplasmic reticulum and mitochondria (Figure 9a); on the other hand, the cell nuclei and the perinuclear membrane are well preserved. Quite similar alterations are also evident in the cells of the capillary endothelium (Figure 9b).

The pattern of cellular damage becomes more marked at later times after irradiation. At 90 min several gaps are detectable in the plasma membrane, while the perinuclear membrane becomes swollen and floccular material can be observed in the intercellular space (Figure 10a); again, parallel changes occur in capillaries (Figure 10b). At 3 h after PDT, several subcellular organelles appear to be poorly identifiable and optically empty and scattered cores of endocytoplasmic coagulation are observed in malignant and endothelial cells (Figure 11a and b). Besides, the alterations of the perinuclear membrane become very pronounced and the nuclear chromatin is pyknotic. The organised subtissular structure is almost completely lost after 8 h (Figure 12a and b): the cellular borders are often undefined, and most cells are completely necrotic with pyknotic nuclei; the residual blood capillaries are stuffed with erythrocytes.

### Discussion

Liposome-incorporated GePc appears to be endowed with a photosensitising activity toward biological substrates at least as high as that exhibited by other widely used tumour photosensitisers, including haematoporphyrin and Zn(II) phthalocyanine, at least as judged by the efficiency of tryptophan photo-oxidation (Jori, 1992*b*). Actually, GePc in the liposomes exists in a purely monomeric state even at relatively large concentrations: the low dielectric constant of the phospholipid bilayer and the presence of bulky axial ligands must drastically weaken the intermolecular hydrophobic interactions which promote the aggregation of macrocyclic porphyrinoid compounds (Reddi & Jori, 1988). Monomeric phthalocyanines are characterised by long-lived triplet states and high quantum yield of photosensitised generation of activated oxygen species. In particular, GePc appears to perform its photosensitising action largely via the intermediacy of singlet oxygen, as shown by (i) the independence of the photo-oxidation rate constant over a wide range of tryptophan concentrations, while alternative photoreaction pathways involving a direct interaction between the excited photosensitiser and the substrate are concentration dependent (Foote, 1976); and (ii) the almost

**Figure 11** Typical micrographs taken 180 min after PDT; the photodamage involves also the nuclei with pyknosis of the chromatin and swelling of the perinuclear membrane (indicated by an arrow in **b**). Bars = 1 $\mu$m. **a**, × 7,500; **b**, × 7,500.

**Figure 12** Typical micrographs obtained 480 min after PDT; both tumour (**a**) and endothelial (**b**) cells are completely necrotic. Bars = 1 $\mu$m. **a**, × 9,000; **b**, × 6,000.

40-fold enhancement of the reaction rate in $D_2O$- as compared with $H_2O$-based media, as one would expect for a predominant singlet oxygen mechanism, since the lifetime of this transient is remarkably longer in deuterated solvents (Gorman & Rodgers, 1992).

In spite of its high reactivity in the electronically excited states, GePc undergoes very little photobleaching, which would cause a permanent chemical modification, even under extreme irradiation conditions. There are divergent opinions as regards the role of dye photobleaching in PDT (Moan & Berg, 1992): the often more rapid photoinduced bleaching of the dye in peritumoral tissues (especially skin) during irradiation facilitates the achievement of a selective tumour damage while precluding a persistent photosensitivity in healthy tissues; on the other hand, photobleaching decreases the effective photosensitiser concentration in the tumour, besides generating photodegradation products whose clearance rate from tissues and possible toxic effects are to be defined. Clearly, a more definite solution of the problems associated with photosensitivity in non-tumoral areas would be represented by a small accumulation in and/or fast elimination of the photosensitiser from normal tissues.

In actual fact, our pharmacokinetic studies indicate a low GePc content in muscle (peritumoral tissue) and skin at all post-injection times examined by us. Even tissues such as liver and spleen, which accumulate large concentrations of GePc, release the phthalocyanine at a relatively fast rate; this observation, coupled with the essentially complete disappearance of GePc from serum within 1 week after administration, should guarantee against long-term undesired dark effects or photo-effects. From this point of view, GePc has a distinct advantage over Photofrin as appreciable amounts of the porphyrin persist in serum, liver and spleen for several weeks (Bellnier *et al.*, 1989). A comparative analysis of the time-dependent biodistribution of a variety of tumour-localising agents suggests that their elimination is facilitated either by the presence of structural features increasing the polarity of the molecule or by the absence of aggregated material (Cuomo *et al.*, 1991). Both these properties are associated with GePc, which is essentially monomeric (at least in the serum) and is made less hydrophobic by the conjugation with two cholesterol moieties.

On the other hand, the selectivity of tumour targeting by GePc, as it is expressed by the ratio of phthalocyanine concentration in the MS-2 fibrosarcoma to the muscle, is similar to that observed for other liposome-delivered phthalocyanines (Cuomo *et al.*, 1991). This could be related to the fact that the presence of two cholesterol moieties brings about only a marginal increase (27% *vs* 20–24%) in the fraction of LDL-bound GePc as compared with other hydrophobic porphyrin or phthalocyanine derivatives (Kongshaug *et al*., 1990; Jori & Reddi, 1993). On the contrary, the addition of cholesterol to the liposome carrier has been shown to increase the amount of Zn(II) phthalocyanine associated with LDL (up to 33%; Ginevra *et al*., 1990). Thus, it appears that manipulation of the photosensitiser molecule has a relatively minor effect on its distribution among the components of the lipoprotein family; rather, the photosensitiser transfer to lipoproteins is mainly controlled by the properties of the delivery system. Even a relatively hydrophilic tetrapyrrole, such as haematoporphyrin, becomes associated with lipoproteins in significant amounts when it is injected *in vivo* after incorporation into suitable liposomes (Beltramini *et al*., 1987).

The combination of a high photosensitising activity and high affinity for the fibrosarcoma explains the excellent PDT efficacy of GePc. All mice that received 1.52 mg $kg^{-1}$ GePc showed an important response of the neoplastic lesion to our irradiation protocol. While similar observations were made for other tumour localisers (Marcus, 1992; Jori, 1992*a*), the tumour-photodamaging process promoted by GePc presents some unique features. In the first place, tumour necrosis develops in a short period of time and reaches a maximal extension at *c*. 3 h after the phototreatment; quite often the extent of PDT-induced necrotic area continues to progress for up to 12–16 h after PDT (see for example Reddi *et al*., 1990). In the present case, the fast propagation rate of the photodamage is likely to reflect the modality of GePc action on the tumour: both malignant cells and the vascular endothelium are heavily affected at the shortest post-irradiation times analysed in our electron microscope studies; in all cases, cell membranes appear to be the main target of the photoprocess while nuclear components are affected only at relatively long times. This would rule out a possible mutagenic action of PDT with the present GePc derivative. On the whole, the time dependence of the development of tumour necrosis in our animal model appears to be correlated with the GePc levels in the tumour, while for more hydrophilic photosensitisers, such as *N*-aspartylchlorine $e_6$, which are known to induce tumour necrosis via a predominantly vascular damage, the response of the tumour to PDT treatment closely reflects the plasma levels of the dye (Gomer & Ferrario, 1990). Previous studies (Zhou, 1989) have shown that liposome-administered photosensitising dyes preferentially affect tumour cells, where they are released by LDLs through receptor-mediated endocytosis. The photodamage of blood capillaries is typically induced by hydrophilic dyes (Reed *et al*., 1988; Henderson & Bellnier, 1989); Kessel *et al*. (1987) proposed that these dyes are largely transported by albumin and deposited in the extracellular matrix. This does not appear to be the case for GePc, since our ultracentrifugation data show that this phthalocyanine is almost exclusively transported by lipoproteins. Therefore, the identification of the factors controlling the modes of tumour photosensitisation by GePc might disclose novel pathways for PDT. It has been often stated that a simultaneous destruction of malignant cells and the tumour vasculature should optimise the outcome of this phototherapeutic modality.

### References

BELLEMO, C., JORI, G., RIHTER, B.D., KENNEY, M.E. & RODGERS, M.A.J. (1992). Si(IV)-naphthalocyanine: modulation of its pharmacokinetic properties through the use of hydrophilic axial ligands. *Cancer Lett.*, **65**, 145–150.

BELLNIER, D.A., HO, Y.K., PANDEY, R.K., MISSERT, J.R. & DOUGHERTY, T.J. (1989). Distribution and elimination of Photofrin II in mice. *Photochem. Photobiol.*, **50**, 221–225.

BELTRAMINI, M., FIREY, P.A., RICCHELLI, F., RODGERS, M.A.J. & JORI, G. (1987). Steady-state and time-resolved spectroscopic studies on the hematoporphyrin–lipoprotein complex, *Biochemistry*, **26**, 6852–6858.

BEN-HUR, E. & ROSENTHAL, E. (1985). The phthalocyanines: a new class of mammalian cell photosensitisers with a potential for cancer phototherapy. *Int. J. Radiat. Biol.*, **47**, 145–147.

BONNETT, R. & BERENBAUM, M. (1990). Porphyrins as photosensitizers. In *Photosensitizing Compounds: Their Chemistry, Biology and Clinical Use*, Bock, G. & Harnett, S. (eds) pp. 40–59. Ciba Foundation Symposium 146, Wiley: Chichester.

CAPRARO, H.-G., SCHIEWECK, K., HILFIKER, R., ISELE, U., VAN HOOGEVEST, P., NAEF, R. & BAUMANN, M. (1994). Synthesis and biological evaluation of new germanium phthalocyanines incorporated into liposomes. Part I: Chemistry. In *Photodynamic Therapy of Cancer*, Jori, G. & Moan, J. (eds) Proceedings EOS/SPIE, Vol. 2078 (in press).

CHAPMAN, M.J. (1986). Comparative analysis of mammalian plasma lipoproteins. In *Methods in Enzymology*, Albens, J.J. & Segret, J.P. (eds) pp. 70–143. Academic Press: London.

CUOMO, V., JORI, G., RIHTER, B., KENNEY, M.E. & RODGERS, M.A.J. (1991). Tumour-localizing and -photosensitizing properties of liposome-delivered Ge(IV)-octabutoxy-phthalocyanine. *Br. J. Cancer*, **64**, 93–95.

DOUGHERTY, T.J. (1987). Photosensitizers: therapy and detection of malignant tumours. *Photochem. Photobiol.*, **45**, 879–889.

FOOTE, C.S. (1976). Photosensitized oxidation and singlet oxygen: consequences in biological systems. In *Free Radicals in Biology*, Vol. II, Pryor, J.A. (ed.) pp. 85–133. Academic Press: New York.

GINEVRA, F., BIFFANTI, S., PAGNAN, A., BIOLO, R., REDDI, E. & JORI, G. (1990). Delivery of the tumour photosensitizer Zn(II)-phthalocyanine to serum proteins by different liposomes: studies *in vitro* and *in vivo*. *Cancer Lett.*, **49**, 59–65.

GOLDSTEIN, J.L., BROWN, M.S., ANDERSON, R.G.W., RUSSELL, D.W. & SCHNEIDER, W.J. (1985). Receptor-mediated endocytosis: concepts emerging from the LDL receptor system. *Ann. Rev. Cell Biol.*, **1**, 1–39.

GOMER, C.J. & FERRARIO, A. (1990). Tissue distribution and photosensitizing properties of mono-L-aspartyl chlorin $e_6$ in a mouse tumour model. *Cancer Res.*, **50**, 3985–3990.

GORMAN, A.A. & RODGERS, M.A.J. (1992). Current perspectives of singlet oxygen detection in biological environments. *J. Photochem. Photobiol., B: Biol.*, **14**, 159–176.

GOTTO, A.M., POWNALL, H.J. & HAVEL, R.J. (1986). Introduction to the plasma lipoproteins. In *Methods in Enzymology*, Segrest, J.P. & Albers, J.J. (eds) pp. 3–41. Academic Press: New York.

HENDERSON, B.W. & BELLNIER, D.A. (1989). Tissue localization of photosensitizers and the mechanism of photodynamic tissue destruction. In *Photosensitizing Compounds: Their Chemistry, Biology and Clinical Use*, Bock, G. & Harnett, S. (eds) pp. 112–130. Ciba Foundation Symposium 146, Wiley: Chichester.

ISELE, U., VAN HOOGEVEST, P., CAPRARO, H.-G. & SCHIEWECK, K. (1993). Pharmaceutical development of CGP 55 847, a liposomal zinc-phthalocyanine formulation using a controlled organic solvent dilution method. In *Photodynamic Therapy of Cancer*, Jori, G. & Moan, J. (eds) Proceedings EOS/SPIE, Vol. 2078 (in press).

JORI, G. (1987). Photodynamic therapy of solid tumours. *Radiat. Phys. Chem.*, **30**, 375–380.

JORI, G. (1992*a*). Low density lipoproteins-liposome delivery systems for tumour photosensitizers *in vivo*. In *Photodynamic Therapy: Basic Principles and Clinical Application*, Henderson, B.W. & Dougherty, T.J. (eds) pp. 173–186. Marcel Dekker: New York.

JORI, G. (1992*b*). Far-red absorbing photosensitizers: their use in the photodynamic therapy of tumours. *J. Photochem. Photobiol., A: Chem.*, **62**, 371–378.

JORI, G. & REDDI, E. (1993). The role of lipoproteins in the delivery of tumour-targeting photosensitisers. *Int. J. Biochem.*, **25**, 1369–1375.

JORI, G., BIOLO, R., MILANESI, C., REDDI, E. & VALDUGA, G. (1991). Zinc(II)-phthalocyanine as a second generation photosensitizing agent in tumour phototherapy. In *Photobiology*, Riklis, E. (ed.) pp. 839–845. Plenum Press: New York.

KESSEL, D., THOMPSON, P., SAATIO, S. & NANTWI, K.D. (1987). Tumour localization and photosensitization by sulphonated derivatives of tetraphenylporphine. *Photochem. Photobiol.*, **45**, 787–789.

KONGSHAUG, M., MOAN, J., RIMINGTON, C. & EVENSEN, J. (1990). Binding of PDT photosensitizers to human plasma studied by ultracentrifugation. In *Photodynamic Therapy of Neoplastic Disease*, Vol. II, Kessel, D. (ed.) pp. 43–62. CRC Press: Boca Raton.

MAGDE, D., BRANNON, J.H., CREMERS, T.L. & OLMSTED, J. (1979). Triphenylmethane dyes as fluorescence standards. *J. Phys. Chem.*, **83**, 666–670.

MARCUS, L. (1992). Photodynamic therapy of human cancer. *Proc. IEEE*, **80**, 869–889.

MOAN, J. & BERG, K. (1992). Photochemotherapy of cancer: experimental research. *Photochem. Photobiol.*, **6**, 931–948.

POLO, L., REDDI, E., GARBO, G.M., MORGAN, A. & JORI, G. (1992). The distribution of the tumour photosensitizers Zn(II)-phthalocyanine and Sn(IV)-etiopurpurin among rabbit plasma proteins. *Cancer Lett.*, **66**, 217–223.

REDDI, E. & JORI, G. (1988). Steady-state and time-resolved spectroscopic studies of photodynamic sensitizers: porphyrins and phthalocyanines. *Rev. Chem. Interm.*, **10**, 214–268.

REDDI, E., RODGERS, M.A.J., SPIKES, J.D. & JORI, G. (1984). The effect of medium polarity on the hematoporphyrin-sensitized photooxidation of L-tryptophan. *Photochem. Photobiol.*, **40**, 415–421.

REDDI, E., LO CASTRO, G., BIOLO, R. & JORI, G. (1987). Pharmacokinetic studies with Zn(II)-phthalocyanine in tumour-bearing mice. *Br. J. Cancer*, **56**, 597–600.

REDDI, E., ZHOU, C., BIOLO, R., MENEGALDO, E. & JORI, G. (1990). Liposome- or LDL-administered Zn(II)-phthalocyanine as a photodynamic agent for tumours. I. Pharmacokinetic properties and phototherapeutic efficiency. *Br. J. Cancer*, **61**, 407–411.

REED, M.W.R., MILLER, F.N., WIEMAN, T.J., TSENG, M.T. & PIETSCH, C.G. (1988). The effect of photodynamic therapy on the microcirculation. *J. Surg. Res.*, **45**, 452–459.

RUDEL, L.L., LEE, J.A., MORRIS, M.D. & FELTS, J.M. (1974). Characterization of plasma lipoproteins separated and purified by agarose-column chromatography. *Biochem. J.*, **139**, 80–85.

SCHERPHOF, G.L., SPANJER, H.H., DERKSEN, J.T.P., LAZAR, G. & ROERDINK, F.H. (1989). Targeting of liposomes to liver cells. In *Drug Carrier Systems*, Roerdink, F.H. & Kroon, A.M. (eds) pp. 281–291. Wiley: Chichester.

SCHIEWECK, K., JORI, G., BATT, E., VAN HOOGEVEST, P. & BIOLO, R. (1990). CGP 48127, a new liposomal Zn(II)-phthalocyanine formulation for photodynamic therapy of tumours. *Abstracts of the 3rd Biennial Meeting of the International Photodynamic Association*, Henderson, B. (ed.) Buffalo, USA. Abstract XXII/4.

SMITH, P.K., KROHN, R.I., HERMANSON, G.T., MALLIA, A.K., GARTNER, F.H., PROVENZANO, M.D., FUJIMOTO, E.K., GOEKE, N.M., OLSON, B.J. & KLENK, D.C. (1985). Measurement of protein using bicinchoninic acid. *Anal. Biochem.*, **150**, 76–85.

SPIKES, J.D. (1986). Phthalocyanines as photosensitizers in biological systems and for the photodynamic therapy of tumours. *Photochem. Photobiol.*, **43**, 691–699.

VAN LIER, J.E. & SPIKES, J.D. (1989). The chemistry, photophysics and photosensitizing properties of phthalocyanines. In *Photosensitizing Compounds: Their Chemistry, Biology, and Clinical Use*, Bock, G. & Harnett, S. (eds) pp. 17–32. Ciba Foundation Symposium 146. Wiley: Chichester.

ZHOU, C. (1989). Mechanisms of tumour necrosis induced by photodynamic therapy. *J. Photochem. Photobiol., B: Biol.*, **3**, 299–318.

ZHOU, C., MILANESI, C. & JORI, G. (1988). An ultrastructural comparative evaluation of tumours photosensitizd by porhyrins administered in aqueous solution, bound to liposomes or to lipoproteins. *Photochem. Photobiol.*, **48**, 487–492.